# Comparative Analysis of the Symbiotic Efficiency of *Medicago truncatula* and *Medicago sativa* under Phosphorus Deficiency

**DOI:** 10.3390/ijms14035198

**Published:** 2013-03-04

**Authors:** Saad Sulieman, Joachim Schulze, Lam-Son Phan Tran

**Affiliations:** 1 Signaling Pathway Research Unit, Plant Science Center, RIKEN Yokohama Institute, 1-7-22, Suehiro-cho, Tsurumi, Yokohama 230-0045, Japan; E-Mail: s.sulieman@psc.riken.jp; 2 Department of Agronomy, Faculty of Agriculture, University of Khartoum, Shambat, Khartoum North 13314, Sudan; 3 Department of Crop Sciences, Section of Plant Nutrition, Georg-August-University of Göttingen, Carl-Sprengel-Weg 1, Göttingen 37075, Germany; E-Mail: jschulz2@gwdg.de

**Keywords:** *Medicago sativa*, *Medicago truncatula*, nitrogen fixation, nodule, symbiosis, phosphorus

## Abstract

Phosphorus (P)-deficiency is a major abiotic stress that limits legume growth in many types of soils. The relationship between *Medicago* and *Sinorhizobium*, is known to be affected by different environmental conditions. Recent reports have shown that, in combination with *S. meliloti* 2011, *Medicago truncatula* had a lower symbiotic efficiency than *Medicago sativa*. However, little is known about how *Medicago*–*Sinorhizobium* is affected by P-deficiency at the whole-plant level. The objective of the present study was to compare and characterize the symbiotic efficiency of N_2_ fixation of *M. truncatula* and *M. sativa* grown in sand under P-limitation. Under this condition, *M. truncatula* exhibited a significantly higher rate of N_2_ fixation. The specific activity of the nodules was much higher in *M. truncatula* in comparison to *M. sativa*, partially as a result of an increase in electron allocation to N_2_*versus* H^+^. Although the main organic acid, succinate, exhibited a strong tendency to decrease under P-deficiency, the more efficient symbiotic ability observed in *M. truncatula* coincided with an apparent increase in the content of malate in its nodules. Our results indicate that the higher efficiency of the *M. truncatula* symbiotic system is related to the ability to increase malate content under limited P-conditions.

Abbreviations

AA: amino acid
ANA: apparent nitrogenase activity
ASN: asparagine
DAT: day after transplanting
DM: dry matter
EAC: electron allocation coefficient
FW: fresh weight
Hup: uptake hydrogenase
Lb: leghemoglobin
N_2_ase: nitrogenase
OA: organic acid
PBM: peribacteroid membrane
PH: phloem
RC: respiratory chain
*Sm*: *Sinorhizobium meliloti*
TNA: total nitrogenase activity
XY: xylem
YEM: yeast extract mannitol

## 1. Introduction

Barrel medic (*Medicago truncatula* Gaertn.) has emerged as a model plant for studying the general biology of legumes and for exploring the genetic and molecular aspects of N_2_-fixing symbiosis in leguminous plants [1–4]. This is due to its relatively small diploid (2*n* = 16) genome (approximately 500 mbp), tractable genetic properties, high level of synteny with several other legumes of interest, and the availability of its genomic sequence [5,6]. Currently, many tools and resources have been developed for this model plant, including ecotype collections (http://www.ars.usda.gov/Main/docs.htm?docid=15140), retrotransposon and fast neutron mutant populations [7], Expressed Sequence Tag (EST) and genespace sequencing information [8,9], transcription factor repertoire-based databases [10,11], and a Gene Expression Atlas [12,13]. Moreover, *M. truncatula* is closely related to the most agricultural important forage legume in the world, *M. sativa* L. (alfalfa, lucerne). *M. sativa* is a perennial crop which is widely cultivated throughout the world and has been a focal point of N_2_ fixation research for many decades [14]. Both *M. truncatula* and *M. sativa* are readily nodulated by the soil bacterium *Sinorhizobium meliloti* (formerly *Rhizobium meliloti*). Today, the *Sm*2011 strain, or its closely related and completely sequenced *S. meliloti* strain 1021, is the most frequently used in studies relating to the biology and genomics of N_2_ fixation [15]. By using the *M. truncatula-Sm*2011 symbiosis as a model system, significant advances have been achieved in understanding the nature of the symbiotic relationship [16,17].

The majority of scientific studies have been directed at dissecting the genetic and molecular bases of the nodulation process in legumes using *M. truncatula* and *Lotus japonicus* as model plants [3,4]. As for *M. truncatula*, there are few published reports focusing on the symbiotic efficiency of Jemalong A17 *vs. S. meliloti* when they are grown under different growth and environmental conditions. We previously documented that the symbiotic association between *M. truncatula* and *Sm*2011 is not as optimum for N_2_ fixation as it is in *M. sativa*[16]. This finding was supported by Terpolilli and co-workers [17], who evaluated the same host plant with the *Sm*1021 of *S. meliloti*. However, the factors responsible for suboptimal N_2_ fixation have not been extensively analyzed. The effectiveness of N_2_ fixation in *M. truncatula*-*Sm*2011, as well as *M. sativa*-*Sm*2011, under phosphorus (P) deficiency has not been examined, despite the fact that P-deficiency is one of the major abiotic stress factors that adversely affects both nodule function and the growth of leguminous plants [18]. Thus, a concerted effort is needed to better understand N_2_ fixation efficiency on a whole-plant basis in *Medicago* plants grown under various environmental conditions, particularly in association with the model organism *S. meliloti.*

Therefore, the main objective of the present study was to extend our previous findings by comparing the effectiveness of N_2_ fixation in *M. truncatula*-*Sm*2011 and *M. sativa*-*Sm*2011 when plants are grown under P-deprivation. The comparative approach was based on the determination of optimal P-levels for each species separately and then using a P-deficiency calculated as 5% of the optimal P-concentration.

## 2. Results

### 2.1. Effect of P-Deficiency on Plant Biomass Production

For comparing the symbiotic efficiency between *M. truncatula* and *M. sativa*, both tested plant species were grown in sand cultures and provided with either 5% (deficient-P) or 100% (control) of their optimum P-requirements. In our calculation, the deficient and optimum P-levels were adjusted according to the daily P-requirement for each species, an important prerequisite that enables the full growth for each species without a possible underestimation for their potential growth. Regardless of P-concentration, shoot and root dry matter (DM) biomasses were significantly higher in *M. sativa* than *M. truncatula*, confirming the relatively low N_2_ fixation efficiency of *Sm*2011 in combination with *M. truncatula* line A17 cv. Jemalong (Table 1) [16]. Decreasing the P-supply from the optimal level resulted in a significant reduction in DM accumulation in the shoot and the total growth for both plant species (Table 1). The higher growth potential of *M. sativa* plants was more sensitive to P-supply and exhibited a stronger decrease in shoot and total DM biomass when P-supply was reduced to 5% of the optimal supply level. Under the sub-optimal P-level used in this study, shoot DM in both species was more affected than root DM. This was particularly evident in *M. sativa*. Consequently, the shoot/root ratio (g g^−1^) was proportionally reduced by decreasing the P-supply (Table 1). Overall, *M. truncatula* reached about a third and a half of the total DM formation compared to *M. sativa* under sufficient and deficient P-supplies, respectively (Table 1).

### 2.2. Effect of P-Deficiency on Nodulation

Nodules first appeared 10 d after inoculation and were clearly visible on plant roots at the time of transplanting to the PVC tubes. Irrespective of the P-supply level, *M. sativa* displayed a greater number of nodules per plant and a higher nodule DM (Table 1). The level of P-deficiency used in this study resulted in a significant reduction in all nodulation parameters, except for the nodule number per plant. Individual nodule DM, as well as total nodule DM, markedly decreased in response to the reduction in P-supply. Overall, both *Medicago* species were similarly affected by the sub-optimal level of P used in this study (Table 1).

### 2.3. Nitrogen Fixation

Table 2 shows the nitrogenase activity in nodules of both species. In general, N_2_ fixation was significantly lower in *M. truncatula* compared to *M. sativa* grown under optimal P-conditions. This is evidenced by the lower apparent nitrogenase activity (ANA) and total nitrogenase activity (TNA) values, irrespective of P-supply. Nodules on both plants appeared healthy and had a reddish color, indicating N_2_ fixation activity. Under P-deficiency, significant reductions were observed in apparent and total values with relatively more decrease in ANA and TNA for *M. truncatla* and *M. sativa*, respectively. A greater reduction in the daily fixed-N was observed in *M. sativa* than in *M. truncatula* in response to sub-optimal P when per plant H_2_ evolution was translated into N_2_ fixation activity (Table 2). These data indicate that the relative higher efficiency of *M. truncatula* nodules under low P-supply was, in part, the result of higher specific activity of electron allocation, *i.e.*, a higher electron allocation coefficient (EAC).

### 2.4. Effect of P-Deficiency on P- and N-Contents

**Figure 1:**
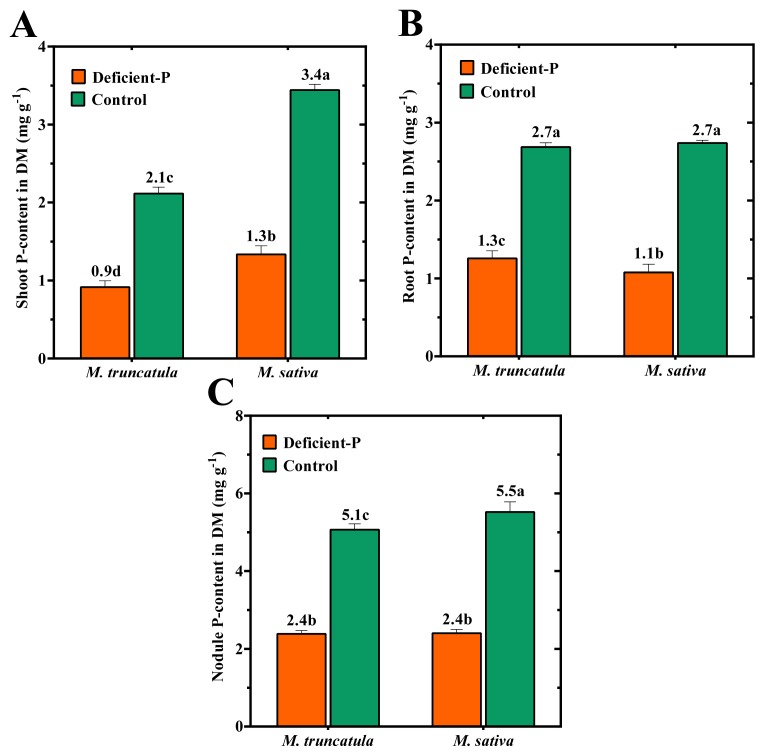
P-content in (**A**) shoots, (**B**) roots and (**C**) nodules of *M. truncatula* and *M. sativa* inoculated with *Sm*2011 and grown over a period of 54 DAT in medium coarse sand supplemented with 100% (Control) or 5% (Deficient-P) of optimal P-level. Data are means of four replicates. Error bars represent standard errors. Data with different letters are significantly different as measured by Tukey’s test (*p* ≤ 0.05). DM, dry matter.

The effect of P-supply on the P-content in different plant organs of both species, relative to DM (mg g^−1^), is illustrated in Figure 1. P-content was higher in nodules than in roots or shoots of both plant species regardless of P-level, indicating that P was preferentially transported into nodules. As expected, a decrease in P-supply resulted in a significant reduction in P-contents in different plant organs, regardless of the tested species. Although P-contents of root and nodule fractions did not differ significantly between *M. sativa* and *M. truncatula* when plants were grown under optimal or sub-optimal P-levels (Figure 1B,C), a striking difference in shoot P-content between the two tested species was observed (Figure 1A).

**Figure 2:**
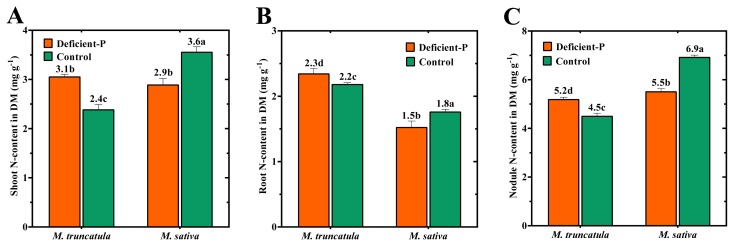
N-content of (**A**) shoots, (**B**) roots and (**C**) nodules of *M. truncatula* and *M. sativa* inoculated with *Sm*2011 and grown over a period of 54 DAT in medium coarse sand supplemented with 100% (Control) or 5% (Deficient-P) of optimal P-level. Data are means of four replicates. Error bars represent standard errors. Data with different letters are significantly different as measured by Tukey’s test (*p* ≤ 0.05). DM, dry matter.

The N-content values for shoots, roots and nodules are shown in Figure 2. Under optimal P-supply, N-content was much lower in shoots and nodules of *M. truncatula* than in *M. sativa*. Under conditions of deficient P, a significant reduction in N-content was observed in *M. sativa*, whereas, *M. truncatula* had a tendency to increase the N-content (Figure 2A,C). This effect was most evident in the shoot and nodule fractions (Figure 2A,C). The shoot and nodule fractions were more negatively affected by P-deficiency in both species compared to the root component.

### 2.5. Major Nodule C- and N-Metabolites

**Figure 3:**
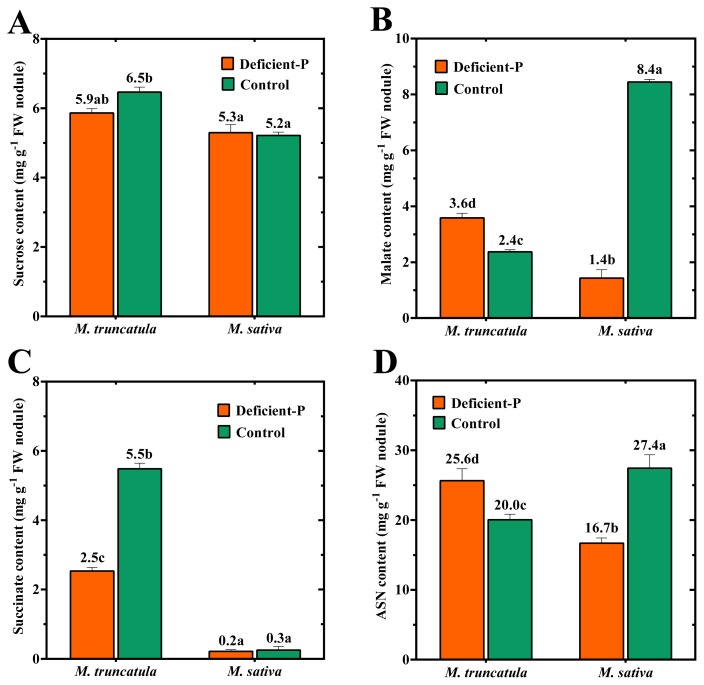
Contents of (**A**) sucrose, (**B**) malate, (**C**) succinate and (**D**) asparagine (ASN) in nodules of *M. truncatula* and *M. sativa* inoculated with *Sm*2011 and grown over a period of 54 DAT in medium coarse sand supplemented with 100% (Control) or 5% (Deficient-P) of optimal P-level. Data are means of four replicates. Error bars represent standard errors. Data with different letters are significantly different as measured by Tukey’s test (*p* ≤ 0.05). FW, fresh weight.

**Figure 4:**
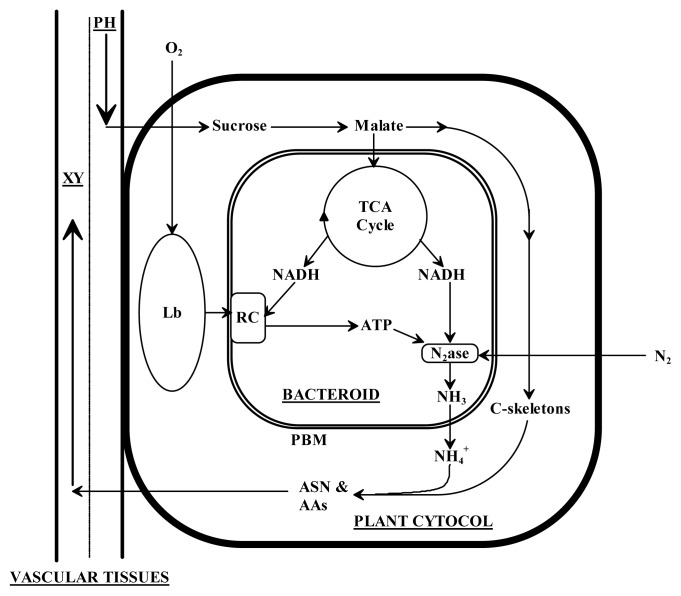
Exchange of metabolites between the host plant and *Sm*2011 during N_2_ fixation in *M. truncatula* and *M. sativa*. Sucrose is delivered to the nodules via the phloem where it is cleaved to malate which is ultimately provided to the bacteroid through the peribacteroid membrane. Within the bacteroid, malate is oxidized by TCA cycle to provide reductants for nitrogenase and for the respiratory chain that fuels nitrogenase with ATP. The presence of leghemoglobin in the host cell controls the concentration of O_2_ available to the respiratory chain in peribacteroid membrane. The bacteroid returns NH_4_^+^ to the host plant which is assimilated into asparagine and other amino acids that are exported from the nodule via the xylem. AAs, amino acids; ASN, asparagine; Lb, leghemoglobin; N_2_ase, nitrogenase; PBM, peribacteroid membrane; PH, phloem; RC, respiratory chain; XY, xylem.

Different patterns in the level of the major C- (sucrose, malate and succinate) and N- metabolites (ASN) were observed in *M. truncatula* and *M. sativa* nodules when plants were grown under P-deficiency compared to when plants were provided with an optimal level of P (Figure 3). Sucrose is known to be the primary source of energy, preferentially imported via the phloem, and is used to sustain nodule function in different legume species (Figure 4) [18]. Nodules of *M. truncatula* had a higher sucrose content compared to those of *M. sativa* (Figure 3A). In response to P-deficiency, sucrose levels in *M. truncatula* nodules decreased slightly compared to *M. sativa* where they were relatively unaffected. In contrast to content of free sucrose in nodules of both species regardless of P-level, the levels of the major organic acids (OAs) and the response to P-level was quite different. Malate, the OA of pivotal importance to legume nodule function was 350% higher in nodules of *M. sativa* than in *M. truncatula* when plants were grown under an optimal P-level (Figure 3B). Under P-deficiency, malate content significantly decreased in nodules of *M. sativa* while the content significantly increased in *M. truncatula*. While malate represents the major OA form in nodules of *M. sativa*, succinate represents the dominant OA in nodules of *M. truncatula*[16]. Succinate content in nodules of *M. truncatula* was significantly reduced when plants were grown under P-deficiency (Figure 3C). In *M. sativa* nodules, no marked difference in succinate content was detected when plants were grown under low P-supply, although overall levels of succinate were much lower than in *M. truncatula* (Figure 3C). The higher per plant and specific (per mg DM nodule) N_2_-fixation activities in *M. sativa* compared to *M. truncatula* was reflected in a higher ASN content in nodules grown under optimal-P (Figure 3D). ASN has been reported to be the predominant amino acid (AA) in nodules of both species [16,19,20]. Under P-deficiency, a significant reduction in the nodule content of ASN in *M. sativa* nodules was observed, while the level of ASN significantly increased in nodules of *M. truncatula* (Figure 3D).

## 3. Discussion

P-deficiency is a major abiotic stress that adversely affects nodulation, N_2_ fixation, and plant productivity throughout the world [18,21,22]. As a macronutrient, P represents a vital component of the symbiotic process due to its role in the generation of the energy that is required for symbiosis to function at a high level [23]. Identification of species or genotypes, or the construction of transgenic plants that possesses higher symbiotic efficiency in low-P soils, is a strategy to overcome this soil constraint.

In the present study, we extended our previous findings by comparing the symbiotic efficiency of *M. truncatula* with *M sativa* under sub-optimal P-conditions using a sand-growing medium. Our results indicated that in combination with *Sm*2011, *M. truncatula* had lower symbiotic efficiency than *M. sativa* when plants were grown in sand culture under optimal P-supply. This finding is reflected in the significantly lower DM accumulation of *M. truncatula* compared to *M. sativa* (Table 1). Quantification of nitrogenase activity and daily fixed-N were consistent with the growth observed in both species (Tables 1 and 2). In agreement with our finding, a study using a hydroponic growth system also reported lower symbiosis efficiency in *M. truncatula* compared to *M. sativa* in plants grown under optimal P-supply [16]. The lower symbiotic efficiency of *M. truncatula* was found to be partly associated with, the limited ability of nodules to export N to the host plant [16]. In the present study, the significantly lower shoot N-content of *M. truncatula* plants, compared to *M. sativa*, in the plants grown in sand under optimal-P strongly supports this interpretation (Figure 2A, control samples). *M. truncatula* is less effective for N_2_ fixation, a situation that might question the popular use of this model plant for genetic and genomic studies relating to N_2_ fixation. However, being a model system, the *M. truncatula*-*Sm*2011 is still the most popular system for conducting research on nodulation and N_2_ fixation [17,24].

**Figure 5:**
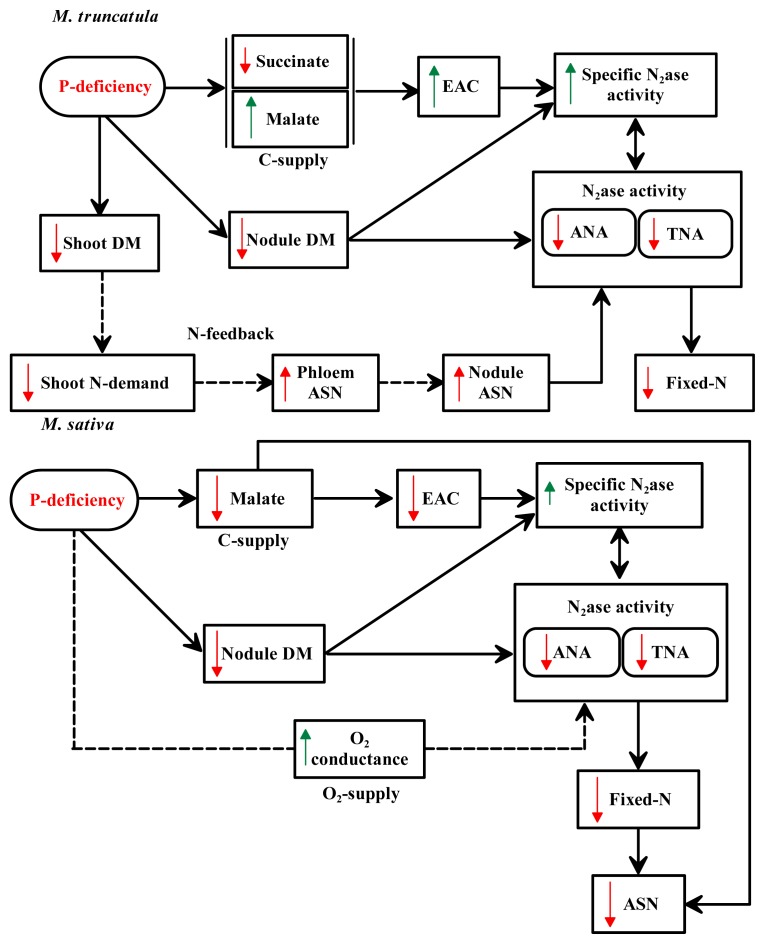
Schematic diagram representing the possible sequence of events leading to decreased N_2_ fixation in nodules of *M. truncatula* and *M. sativa* plants subjected to P-deficiency. The model indicates the effect of C-supply, N-feedback, and O_2_-supply on nitrogenase activity. P-deficiency is expected to affect nodule C-metabolism by shifting the content of OAs in both plant species, *i.e.*, malate and succinate. As a result, the activity and per unit fixed-N would increase and decrease in nodule of *M. truncatula* and *M. sativa*, respectively. Under such conditions, the reduced-N demand in *M. truncatula* may result in a feedback mechanism with ASN suggested to be the shoot-N signal. In *M. sativa*, a higher O_2_ uptake would expect to have a regulatory implication. ANA, apparent nitrogenase activity; ASN, asparagine; EAC, electron allocation coefficient; N_2_ase, nitrogenase; TNA, total nitrogenase activity. Red and green arrows indicate inhibition and stimulation, respectively. See text for more details.

Under conditions of P-deficiency, *M. truncatula* displayed a higher symbiotic efficiency than *M. sativa*. This finding was reflected in the lower reduction in DM accumulation and the lower sensitivity of nitrogenase in *M. truncatula* than in *M. sativa* when plants were grown under conditions of P-deficiency (Tables 1 and 2). For *M. sativa*, the negative effect of P-deficiency was more pronounced in TNA, as well as the daily fixed-N amount (Table 2), while the degree of reduction in nodule number and weight were relatively similar to *M. truncatula* (Table 1). Additional analyses indicated that the higher symbiotic efficiency observed in *M. truncatula* under P-deficiency is probably related to nitrogenase activity. When the reasons behind the higher level of symbiotic efficiency of *M. truncatula* compared to *M. sativa* in plants grown under P-deficiency are examined, it is evident that the enhanced symbiosis is mainly due to the increased specific activity of nodules (N_fixed_ per unit nodule biomass), partially as a result of a higher relative efficiency in electron allocation to N_2_*versus* H^+^ (Figure 5). While a significant increase in EAC was detected in *M. truncatula*, a significant reduction was observed in *M. sativa* (Table 2) (Figure 5). The shifts in nodule-specific activities for both species were most likely related to nodule C-metabolism, *i.e.* capacity to produce OA. Malate played a pivotal role in determining the symbiotic efficiency and the level of nitrogenase activity in both of the examined species (Figure 5). Under P-deficiency, the content of this OA was significantly reduced in *M. sativa* nodules; a condition that would result in a sharp decrease in energy available for nitrogenase activity (Figure 5). Malate is the principle source for providing energy and reductants for nitrogenase activity and C-skeletons for fixed-N assimilation, nodule growth and maintenance (Figures 4 and 5) [25,26]. Although a significant decrease in succinate was observed in *M. truncatula*, a concomitant increase in malate content was detected in the nodules of plants grown under P-stress (Figures 3 and 5). Succinate represents a major portion of the total OAs detected in nodules of *M. truncatula* under conditions of optimal-P [16]. Accordingly, the higher efficiency in *M. truncatula* nodules under P-stress is one of the reasons that nodule C-metabolism is shunted towards OAs, namely malate formation (Figure 5).

Indeed, exposure of both species to P-deficiency had a dramatic effect on C- and N-metabolism, and, as a result, symbiotic efficiency (Figure 5). While the content of ASN significantly decreased in nodules of P-deficient *M. sativa* plants, a significant accumulation of this amide was detected in nodules of *M. truncatula* (Figure 3D). ASN has been identified as the primary assimilation product from N_2_ fixation in temperate legumes and the predominant N-transported substance in several plants that have nodules with indeterminate growth, including *Medicago* (Figure 4) [18,26]. Thus, the significant reduction in ASN in nodules of *M. sativa* under P-deficiency was mirrored in the reduced amount of the daily fixed-N (Table 2; Figure 5). The close association between ASN content in nodules and the fixed N amount in *M. sativa* was previously reported [20,27]. The lower ASN content in nodule tissues of *M. sativa* was brought about by the insufficient amount of C available for amide formation, as a direct result of low malate (Figure 5). In contrast, the significantly higher content of ASN in *M. truncatula* nodules of plant grown under P-deficiency was observed in our study and other reports to be of a shoot origin (Figure 5) [18,26]. This amide consistently accumulates in different legume organs in response to various stresses, such as drought [28], salt stress [29], P-deficiency [30], defoliation [31] and nitrate application [18]. It is widely accepted that nitrogenase activity is regulated by an N-feedback mechanism, *i.e.* N-demand [28,32,33]. In this type of systemic control, specific potential phloem-translocated signals are required to be sent back to the nodule to modulate their activity [34,35]. ASN has been identified to be among the prime candidate signals suspected to downregulate the nodule nitrogenase activity (Figure 5) [26,30]. This premise was supported by our previous study in which intact *M. truncatula* plants were fed with a high concentration of this amide [18]. The study indicated that exogenous feeding of phloem with 3.0 mM ASN resulted in a greater increase in content of ASN in nodules while concomitantly reducing nitrogenase activity. The shoot and nodule N-contents apparently support this interpretation for both species (Figure 2A,C). No indications of N-feedback were observed in our measurements of *M. sativa* in the present study that could explain the observed significant reductions in N_2_ fixation observed in plants grown under P-deficiency. Alternatively, several reports have indicated higher O_2_ permeability and conductance in the nodules of *M. sativa* under P-deficiency which might have regulatory implications [23,36].

## 4. Experimental Section

### 4.1. Plant Materials and Growth Conditions

*M. truncatula* (Gaertn.) cv. “Jemalong A17” (barrel medic) and *M. sativa* (L.) cv. “Saranac” (alfalfa) were used throughout the study in combination with *Sm*2011. Seeds of *M. truncatula* were soaked in concentrated H_2_SO_4_ for five minutes with intermittent gentle shaking. The acid was decanted and the seeds were rinsed thoroughly five times with sterile water. Subsequently, seeds were placed in 5% sodium hypochlorite for three minutes, followed by rinsing eight times with sterile water immediately after decanting the bleach. Following scarification and surface sterilization, seeds were placed in sterile water at 4 °C for 48 h. Subsequently, seeds were sown on sterilized fine quartz sand (Ø = 0.1–0.5 mm, Quarzsandwerke Weferlingen, Germany). *M. sativa* seeds were surface-sterilized with 70% ethanol for 10 min, washed in sterile water and germinated on sterilized fine quartz sand supplied with tap water to 70% of the maximum water-holding capacity. *M. truncatula* and *M. sativa* plants were maintained in a controlled growth chamber with a 16/8 h day/night cycle, approximately 25/18 °C day/night temperature, 70% relative humidity and 360 μmol m^−2^ s^−1^ photosynthetic active radiation. For inoculation, *Sm*2011 was grown in yeast extract mannitol (YEM) broth (mannitol 10 g L^−1^; yeast extract 0.5 g L^−1^; K_2_HPO_4_ 0.5 g L^−1^; MgSO_4_.7H_2_O 0.2 g L^−1^; NaCl 0.1 g L^−1^; pH 6.8, for 72 h at 28 °C), to an approximate cell density of 10^−7^. Fifty mL of bacterial suspension which was diluted with water to a final concentration of optical density at 600 = 0.1 was added after the seeds were sown in the sand. The first nodules became visible about 10 d after inoculation.

*M. truncatula* and *M. sativa* plantlets inoculated with *Sm*2011 were transferred to sterilized medium coarse quartz sand in PVC tubes (one plant per tube). Two hundred mL of a nutrient solution was supplied daily to the sand cultures. The nutrient solution had the following composition: 700 μM K_2_SO_4_; 500 μM MgSO_4_; 800 μM CaCl_2_; 4.0 μM H_3_BO_3_; 0.1 μM Na_2_MoO_4_; 1.0 μM ZnSO_4_; 2.0 μM MnCl_2_; 0.2 μM CoCl_2_; 1.0 μM CuCl_2_ and 10.0 μM FeNaEDTA (ferric monosodium salt of ethylenediamine tetraacetic acid). The pH was buffered with 2 mM MES [2-(*N*-morpholino) ethane-sulfonic acid] and adjusted to 6.5 with KOH. Urea was added to the nutrient solution at a final concentration of 0.2 mM for the first 10 DAT. Thereafter, plants did not receive an external source of N. P-levels (deficient *vs.* optimum) were designed according to the calculated requirements for each species independently. The optimal concentration of P (designed as 100%) was first determined, and used as the basis to calculate a deficient P-level (5% of the optimum level). In the calculation of the optimal (100%) P-requirement for each species, seed P-content, plant growth rate, and plant age were considered. Based on preliminary experiments, the plants’ growth for both species was in an exponential manner and the P-requirement follows the same pattern with time. In our calculations, the assumption was made that plants of *M. sativa* and *M. truncatula* supplied with a sufficient level of P have 3.8 and 2.5 mg P per g shoot DM, respectively [18,23]. Accordingly, the 5% deficient levels were estimated to be around 0.19 and 0.13 mg P per day for *M. sativa* and *M. truncatula*, respectively. KH_2_PO_4_ was the source of P in the experiment. Plants were harvested at 54 DAT, and nodule number and shoot, root and nodule dry matter were determined.

### 4.2. Measurements of Nitrogenase Activity

The measurement of H_2_ evolution is an indirect parameter for the determination of N_2_-fixation activity of legume nodules. The *Sm*2011 (hup^−^) that was used in the study does not have an uptake hydrogenase [16]. For H_2_ evolution measurements, the sealed root/nodule compartment was connected to an open-flow gas exchange measurement system that allowed for the application of a mixture of N_2_/O_2_ (80/20, *v*/*v*) to the root/nodule compartment. For the measurements, the level of the nutrient solution was reduced to the lower parts of the PVC tubes. A gas flow of 200 mL min^−1^ (about 1.2 vols min^−1^) was applied to the root compartment. A subsample (100 mL min^−1^) of the outflowing gas was taken, dried (ice trap and MgClO_4_) and passed through an H_2_ analyzer (S121 Hydrogen analyzer, Qubit Systems, Canada). When a stable H_2_ outflow from the root/nodule compartment was reached, this value was taken as ANA. To obtain the information on the relative efficiency (electron allocation between N_2_ and H^+^), the inflow air composition was subsequently switched to Ar/O_2_ (80/20, *v*/*v*). Argon is inert to nitrogenase and thus the whole electron flow is diverted to H^+^. Consequently, H_2_ evolution under argon represents the total enzyme activity (TNA). The peak value, taken approximately 5 min after switching to Ar/O_2_, was recorded as the TNA value. EAC of nitrogenase activity was calculated as 1–(ANA/TNA) [25,37,38]. In preliminary experiments, the validity of the H_2_ evolution measurements was tested by parallel ^15^N_2_-application [16]. The amount of fixed nitrogen per unit time and per plant or nodule was calculated on the basis of the ANA and TNA measurements [37]. ANA, TNA, and the EAC were measured at the end of the experimental period.

### 4.3. HPLC Analyses

For the detection of ASN, sucrose and OAs (malate and succinate), nodules were picked from intact plant roots, immediately frozen in liquid N_2_ and stored at −20 °C until analyzed. Frozen nodule samples were ground to a fine powder in liquid N_2_ using a mortar and pestle. Approximately 0.5 mg of each sample was extracted with 3 mL of 50% ethanol (*v*/*v*) for 20 min at 50 °C in a water bath. The solution from each sample was subsequently centrifuged at 4 °C for 20 min at 8000 rpm (6810 g). The supernatant was immediately used for HPLC analyses after filtration (0.45 mm). ASN content was detected with a fluorescence detector (Waters, Milford, MA, USA) after precolumn derivatization by *o*-phthaldialdehyde [39]. The detection of sucrose was done with a refractometer (Knauer, Berlin, Germany), while the determination of malate and succinate was carried out using a photodiode array detector 996 (Waters, Milford, MA, USA).

### 4.4. Determination of P- and N-Contents

Dry matters from various plant organs (shoot, root and nodules) were ground to a fine powder in a pebble mill and used for determination of P- and N-contents. The plant samples were weighed, ground, and the sub-samples (0.3 g) digested with a mixture of HNO_3_ and H_2_O_2_ (30%) in a volumetric ratio (4:2) in a microwave oven. Phosphate in the extract was measured calorimetrically, as previously described [40]. Nitrogen was determined using a C/N analyzer (NA 2500, CE-Instruments, Milano, Italy) and a mass spectrometer (Finnigan MAT, model 252, Bremen, Germany).

### 4.5. Statistical Analysis

Data were subjected to an analysis of variance using two-way ANOVA procedure of the Sigmastat analytical software (version 3.5, Systat software, Inc.: San Jose, CA, USA). In the case of homogeneous sample variances, mean separation procedures were carried out using the Tukey’s test.

## 5. Conclusions

The present study has provided a foundation for in-depth molecular studies of the relationship between P-deficiency and symbiotic efficiency in *M. truncatula*, with the ultimate aim of developing efficient N_2_-fixing leguminous cultivars. Our data indicate that nodules formed in the *M. truncatula*-*Sm*2011 symbiotic relationship have the ability to produce OAs, particularly malate, when plants are grown under conditions of P-deficiency resulting in an increase in the specific activity of nodules. Based on this premise, an improvement in malate formation in *M. sativa* may represent a promising strategy for improving N_2_-fixation efficiency in alfalfa and other leguminous plants.

## Figures and Tables

**Table 1:** Dry matter production and nodulation levels in *Medicago truncatula* and *Medicago sativa* inoculated with *Sm*2011 and grown over a period of 54 DAT (day after transplanting) in medium coarse sand supplemented with 100% (Control) or 5% (Deficient-P) of optimal P-level. Data presented are the means ± SE of four replicates.

	*M. truncatula*		*M. sativa*	
		
	Deficient-P	Control	Deficient-P	Control
DM (g plant^−1^)				
Shoot	0.17 ± 0.01 ^b^	0.71 ± 0.11 ^c^	0.40 ± 0.06 ^b^	2.67 ± 0.23 ^a^
Root	0.20 ± 0.01 ^b^	0.48 ± 0.01 ^b^	0.43 ± 0.07 ^b^	1.62 ± 0.18 ^a^
Total	0.39 ± 0.02 ^b^	1.22 ± 0.10 ^c^	0.85 ± 0.13 ^b^	4.33 ± 0.39 ^a^
Shoot/Root (g g^−1^)	0.84 ± 0.02 ^b^	1.50 ± 0.27 ^a^	0.93 ± 0.02 ^b^	1.67 ± 0.11 ^a^
Nodule number plant^−1^	13.0 ± 1.6 ^a,b^	17.0 ± 1.3 ^b^	21.0 ± 3.2 ^a^	27.0 ± 3.7 ^a^
Nodule DM (mg plant^−1^)	11.8 ± 1.6 ^b^	29.4 ± 2.9 ^c^	16.1 ± 1.8 ^b^	41.8 ± 1.8 ^a^
Individual nodule DM (mg)	0.9 ± 0.1 ^b^	1.7 ± 0.1 ^a^	0.9 ± 0.2 ^b^	1.6 ± 0.2 ^a^

Data with different letters are significantly different as measured by Tukey’s test (*p* ≤ 0.05). DM, dry matter.

**Table 2:** Nitrogenase activity, electron allocation, and N_2_ fixation in *M. truncatula* and *M. sativa* inoculated with *Sm*2011 and grown over a period of 54 DAT in medium coarse sand supplemented with 100% (Control) or 5% (Deficient-P) of optimal P-level. Data presented are the means ± SE of four replicates.

	*M. truncatula*		*M. sativa*	
		
	Deficient-P	Control	Deficient-P	Control
Nitrogenase activity:				
ANA (μmol H_2_ plant^−1^ h^−1^)	0.36 ± 0.04 ^d^	0.78 ± 0.03 ^c^	1.85 ± 0.08 ^b^	2.99 ± 0.24 ^a^
TNA (μmol H_2_ plant^−1^ h^−1^]	0.99 ± 0.08 ^c^	1.66 ± 0.08 ^c^	3.53 ± 0.30 ^b^	7.40 ± 0.49 ^a^
EAC	0.63 ± 0.02 ^d^	0.52 ± 0.01 ^a,c^	0.47 ± 0.03 ^b^	0.60 ± 0.02 ^a^
Fixed-N per plant (mg N 24 h^−1^)	0.14 ± 0.01 ^c^	0.20 ± 0.01 ^c^	0.38 ± 0.05 ^b^	0.99 ± 0.08 ^a^
Specific fixed-N (μg N mg nodule^−1^ h^−1^)	12.96 ± 2.71 ^c^	6.94 ± 1.01 ^b^	25.62 ± 7.08 ^a^	23.63 ± 1.66 ^a^

Data with different letters are significantly different as measured by Tukey’s test (*p* ≤ 0.05). ANA, apparent nitrogenase activity; EAC, electron allocation coefficient; Fixed N per plant, total fixed-N per plant per day; Specific fixed N, N_fixed_ per unit nodule biomass; TNA, total nitrogenase activity.
